# Signaling pathways in the development of infantile hemangioma

**DOI:** 10.1186/1756-8722-7-13

**Published:** 2014-01-31

**Authors:** Yi Ji, Siyuan Chen, Kai Li, Li Li, Chang Xu, Bo Xiang

**Affiliations:** 1 Division of Oncology, Department of Pediatric Surgery, West China Hospital of Sichuan University, Chengdu 610041, China; 2 Pediatric Intensive Care Unit, West China Hospital of Sichuan University, Chengdu 610041, China; 3 Division of Oncology, Department of Pediatric Surgery, Children’s Hospital of Fudan University, Shanghai 201102, China; 4 Laboratory of Pathology, West China Hospital of Sichuan University, Chengdu 610041, China

**Keywords:** Infantile hemangioma, Neovascularization, Angiogenesis, Vasculogenesis

## Abstract

Infantile hemangioma (IH), which is the most common tumor in infants, is a benign vascular neoplasm resulting from the abnormal proliferation of endothelial cells and pericytes. For nearly a century, researchers have noted that IH exhibits diverse and often dramatic clinical behaviors. On the one hand, most lesions pose no threat or potential for complication and resolve spontaneously without concern in most children with IH. On the other hand, approximately 10% of IHs are destructive, disfiguring and even vision- or life-threatening. Recent studies have provided some insight into the pathogenesis of these vascular tumors, leading to a better understanding of the biological features of IH and, in particular, indicating that during hemangioma neovascularization, two main pathogenic mechanisms prevail, angiogenesis and vasculogenesis. Both mechanisms have been linked to alterations in several important cellular signaling pathways. These pathways are of interest from a therapeutic perspective because targeting them may help to reverse, delay or prevent hemangioma neovascularization. In this review, we explore some of the major pathways implicated in IH, including the VEGF/VEGFR, Notch, β-adrenergic, Tie2/angiopoietins, PI3K/AKT/mTOR, HIF-α-mediated and PDGF/PDGF-R-β pathways. We focus on the role of these pathways in the pathogenesis of IH, how they are altered and the consequences of these abnormalities. In addition, we review the latest preclinical and clinical data on the rationally designed targeted agents that are now being directed against some of these pathways.

## Background

**Figure 1:**
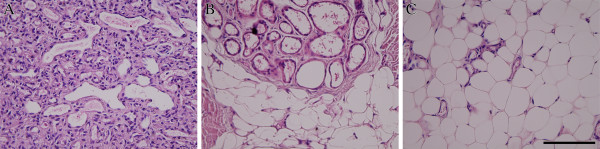
**Hematoxylin and eosin (H&E) stained sections of proliferating, involuting and involuted phases of IH.** The proliferating phase is characterized by densely packed tumor cells that form immature vessels **(A)**. In the involuting phase, disorganized vasculature consists of flat endothelium and pericytes **(B)**. The tumor is replaced by fat and/or connective tissues in the involuted phase **(C)**. Scale bar = 100 μm.

Infantile hemangioma (IH) is a common disorder in infancy, with an estimated prevalence of 5 to 10%. If left untreated, these tumors are characterized by a rapid growth phase during the first year of life, followed by slow involution, which may continue until the age of 10–12 years (Figure 1) [1,2]. However, some IHs will leave residual changes, such as telangiectasias, fibro-fatty tissue, scars, excessive atrophic skin and pigment changes. In 10% of cases, IHs grow dramatically and destroy tissue, impair function or even threaten life [3]. The standard treatment options for IH include corticosteroids or surgical excision, and the options in life- or sight-threatening cases include treatment with vincristine, interferon or cyclophosphamide. Unfortunately, none of these therapeutic modalities are ideal due to restrictions or potential serious side effects [4-7]. β-blockers have recently been introduced as a safe and effective treatment for IH [8-11]. However, their use is not without risk, and not all tumors respond to these drugs [12,13]. These issues have spurred extensive research to clarify the signaling pathways implicated in hemangioma neovascularization in the hope that a greater understanding of its molecular pathogenesis will reveal new strategies to tackle IH.

The initial histochemical work of Mulliken and Glowacki [14], examining endothelial cell (EC) morphology, shed light on the cellular components of IH. In the past decade, hemangioma-derived progenitor/stem cells (HemSCs), mesenchymal stem cells (Hem-MSCs), endothelial progenitor cells (HemEPCs), ECs (HemECs) and perivascular cells (Hem-pericytes), all of which comprise the IH, have been isolated (Table 1) [15-18]. In general, CD133 was used as a stem cell biomarker for the isolation of HemSCs from IH tissues. HemEPCs were purified from HemSCs based on expression of the EC marker CD31. In contrast, Hem-MSCs didn’t express CD31 or CD34. In IH tissues, CD133 expression was found to be located in both perivascular region and endothelium [19]. Therefore, HemSCs may contain both of Hem-MSCs and HemEPCs. Studies from different groups have demonstrated that HemSCs have the ability to self-renew and can differentiate into endothelium, adipocytes and pericytes in vitro [15,20]. When implanted subcutaneously into nude mice, HemSCs can produce human glucose transporter-1 (GLUT-1) positive microvessels at 7–14 days [15,20-22].

**Table 1:** Cellular components isolated from IH

**Cell type**	**Abbreviation**	**Cell marker**	**Characteristics**
Hemangioma-derived endothelial cell	HemEC	CD31/PECAM-1, vWF, E-selectin, VEGFR-2, Tie-2 and VE-cadherin	Immature endothelial cells; Clonal expansion; Increased proliferation, migration, tumor formation and survival ability.
Hemangioma-derived endothelial progenitor cell	HemPEC	CD133*, VEGFR-2, CD34, CD31, CD146, VE-cadherin and vWF	Immature endothelial cells; Increased adhesion, migration and proliferation in the presence of endostatin or VEGF.
Hemangioma-derived mesenchymal stem cell	Hem-MSC	SH2(CD105), SH3, SH4, CD90, CD29, α-SMA and CD133	Multilineage differentiation: adipogenic, osteoblastic and myoblastic differentiation
Hemangioma-derived stem cell	HemSC	CD90, CD133, VEGFR-1, VEGFR-2, neuroplin-1 and CD146	Multilineage differentiation: ECs, neuronal cells, adipocytes, osteocytes and chondrocytes; Form hemangioma-like Glut-1^+^ blood vessels in nude mice.
Hemangioma-derived pericyte	Hem-pericyte	PDGFR-β, neural glial antigen-2, desmin, calponin, smooth muscle 22α, smooth muscle α-actin, α-SMA, smooth muscle myosin heavy chain and CD90	Increased proliferation ability; Reduced contractility; Diminished ability to stabilize blood vessels in IH.

*CD133, a pentaspan membrane protein, is used as a stem cell biomarker for the isolation of progenitor/stem-like cells from IH tissues. CD133 is also responsible for self-renewal, tumorigenesis, metabolism, differentiation, autophagy, apoptosis and regeneration [23]. However, little is known about its biological functions in the development of IH.

We now recognize that IH may be not only a disorder of angiogenesis (i.e., the sprouting of new vessels from existing ones) but also – at least in part – a disorder of vasculogenesis (i.e., the de novo formation of new blood vessels from stem cells) [20,24,25]. Improved knowledge of the signaling pathways that regulate angiogenesis and vasculogenesis has led to the identification of several possible therapeutic targets that have driven the development of molecularly targeted therapies. Because many of the signaling pathways are implicated in the pathogenesis of various tumor types, insight gained from these studies will enable the development of target-specific drugs, not only for IH but also for malignant vascular tumors. This review will highlight the most important of these findings. Although the signaling pathways involved in the development of IH are described separately below, there are numerous interactions among them, indirectly reflecting the complexity of IH pathogenesis.

### VEGF/VEGFR pathway

The human vascular endothelial growth factor (VEGF) family consists of VEGF-A, VEGF-B, VEGF-C, VEGF-D and placental growth factor (PIGF). These growth factors play pivotal roles in embryonic development and angiogenesis-dependent disease [26]. Many reports have confirmed that excessive VEGF expression in IH tissue parallels the proliferating phase of its growth. Conversely, in the involuting phase, VEGF expression rapidly decreases, and many angiogenesis inhibitors become prominent [21,27,28].

The functions of the different VEGF family members are determined by their receptor specificity. Two receptors for VEGF are members of the tyrosine-kinase family and conserved in ECs. These VEGF receptors (VEGFR) are VEGFR-Flt-1 (VEGFR-1) and VEGFR-Flk-1/KDR (VEGFR-2). VEGFR-1 and VEGFR-2 are located on ECs, bone-marrow derived hematopoietic cells and tumor cells, etc. [26,29]. The expression of these receptors is low in normal tissues and only upregulated during the development of those pathological states when neovascularization occurs [30]. Another receptor, VEGFR-3, is primarily expressed in lymph nodes and tumor blood vessels [31,32]. Neuropilin-1 and neuropilin-2 were discovered as coreceptors that that enhanced the binding and effectiveness of the VEGF stimulation of their receptors [33].

#### Upregulated autocrine VEGF-A/VEGFR-2 loop in HemEC

One of the most intensely studied factors involved in angiogenesis and vasculogenesis is VEGF-A. VEGFR-2 is known to mediate the majority of the downstream angiogenic effects of VEGF-A, including microvascular permeability, EC proliferation, migration and survival [34]. Upon the activation of VEGFR-2 in ECs, three major secondary messenger pathways trigger multiple downstream signals that promote angiogenesis. These pathways are the following: the mitogen-activated protein/ERK kinase (MEK)/extracellular signal-regulated kinase (ERK) cascade, the phosphatidylinostitol-3 kinase (PI3K)/serine-threonine protein kinase/Akt cascade and the phospholipase C-γ/intracellular Ca^2+^/(protein kinase C (PKC) cascade [35,36]. The genetic deletion of VEGF-A or its primary signaling receptor VEGFR-2 results in early embryonic lethality, associated with a near-complete block of hematopoietic and vascular development [37].

**Figure 2:**
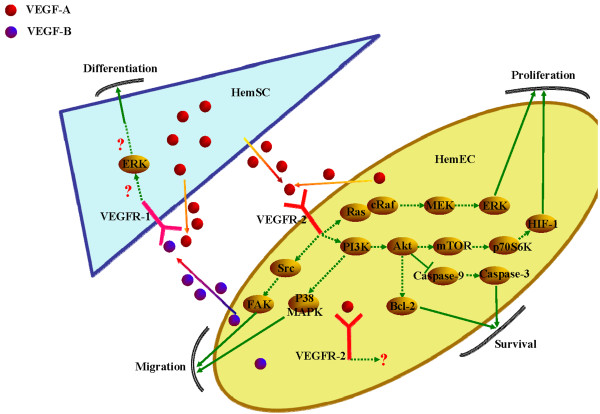
**The VEGF signaling pathway in HemECs and HemSCs.** Upon ligand binding, VEGF receptors dimerize, leading to the phosphorylation of different tyrosine residues. Phosphorylation in turn elicits differential downstream signaling events.

High-VEGFR-2 cells are well documented to exhibit a higher capacity of self-renewal and superior growth in vitro and in vivo compared with a low-VEGFR-2 cell population [33,38]. HemECs demonstrate the phenotype of a constitutively active autocrine VEGF-A/VEGFR-2 loop (Figure 2), which renders the cells more sensitive to paracrine/external stimulation by VEGF-A and results in the increased proliferation and migration of cells and tumor formation [39,40]. These characteristics likely result from the genetic instability of HemECs as somatic missense mutations in the kinase insert of the VEGFR-2 gene have been found in IHs [41]. In addition, imbalances in gene expression have been reported in mesenchymal compartments compared to normal tissues, suggesting a possible reciprocal interaction between HemECs and the surrounding cells [42-44]. Alternatively, HemECs may originate from progenitor/stem-like cells, which are known to display robust proliferative and clonogenic capabilities and to express high levels of VEGF-A [15,21]. The expression level of VEGF is also increased in HemECs, although this increase is not dramatic [40]. Finally, COSMC was reported to be overexpressed in proliferating IHs, with an association with the enhanced VEGF-mediated phosphorylation of VEGFR-2 and its downstream signaling [45].

**Figure 3:**
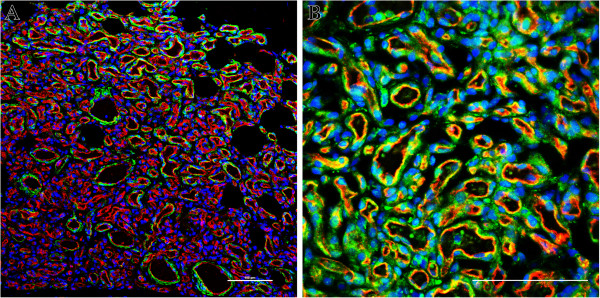
**Double immunofluorescence staining of IH tissues. (A)**, Proliferating phase IH tumor section stained for endothelial maker CD31 (red), smooth muscle marker α-SMA (green) and nuclei (blue) (laser fluorescent confocal microscopy). **(B)**, Proliferating phase IH tumor section stained for CD31 (red), VEGF-A (green) and nuclei (blue) (fluorescent microscopy). The nuclei are stained with DAPI. Scale bars are 100 μm.

The abnormal activation of VEGFR-2 on the cell surface may also be beneficial to the survival of HemECs as VEGF-A plays a critical role in protecting ECs against apoptotic cell death [46,47]. In addition, this inhibition of EC apoptosis can improve angiogenesis and vasculogenesis in patients with ischemia [30]. We recently indicated that maintaining Bcl-2 expression via VEGF-A/VEGFR-2 signaling in primary HemECs blocked the cells from apoptotic death in the absence of external VEGF-A. Moreover, the inactivation of PI3K/Akt suppressed the VEGF-A/VEGFR-2-mediated anti-apoptotic effect and unleashed the inhibitory effect of VEGF-A/VEGFR-2 signaling over the reduction of Bcl-2 expression, thereby amplifying the activation of the caspase cascade [48]. These findings suggest that HemECs may be able to adapt to the abnormal physical environment of the tumor by undergoing a form of reprogramming that involves an increase in apoptosis resistance and by up-regulating a VEGF autocrine survival feedback loop to sustain these effects and stabilize the aberrant phenotype. Moreover, recent research efforts revealed that pericytes, in addition to producing VEGF-A that acts in a paracrine fashion (Figure 3), can stimulate the autocrine expression of VEGF-A by tumor ECs, both of which could lead to a general suppression of EC apoptosis [49]. Interestingly, HemSCs and Hem-pericytes also secrete high levels of the angiogenic VEGF-A [21,42]. Thus, various combinations of strategies, including the development of novel potent tyrosine kinase inhibitors against VEGFR-2 and potential to abrogate its downstream pathways, can be investigated to achieve synergistic effects on HemEC apoptosis and therefore on hemangioma regression.

#### Discrepancy of VEGFR-1 signaling in HemSCs and HemECs

In contrast to other VEGFR genes, VEGFR-1 expresses two types of mRNA, one for a full-length receptor and another for a soluble short protein known as soluble VEGFR-1 (sFlt-1). The binding-affinity of VEGFR-1 for VEGF-A is one order of magnitude higher than that of VEGFR-2, whereas the kinase activity of VEGFR-1 is about ten-fold weaker than that of VEGFR-2. Therefore, VEGFR-1 is considered a negative regulator of angiogenesis and vasculogenesis during development [50]. VEGFR-1^-/-^ mice show an overabundance of blood vessels and overgrowth of immature ECs, similarly to those features observed in IH [51].

A reduction of VEGFR-1 expression has been implicated in the proliferation of infantile HemECs and tissue [40,52]. The mechanism for this low expression in HemECs was shown to be the sequestration of *β*1-inergrin in a multiprotein complex composed of tumor endothelial marker-8 (TEM8) and VEGFR-2, which inhibits nuclear factor in activated T cells (NFAT)-mediated VEGFR-1 transcription [40]. However, VEGFR-1 is relatively over-expressed in HemSCs [53]. The involvement of VEGFR-1 in the progression of IH could involve at least one mechanism: the activation of HemSC function, with a subsequent increase in vasculogenesis. VEGF-A, either endogenous or exogenous, significantly induces VEGFR-1-mediated ERK1/2 phosphorylation in HemSCs and promotes the differentiation of HemSCs to HemECs (Figure 2). Moreover, VEGF-B, which is the specific ligand for VEGFR-1, is highly expressed in HemECs and induces similar effects [53]. These results clearly indicate that not only the paracrine function of VEGF-B from HemECs but also the persistent autocrine signaling through the VEGF-A/VEGFR-1 loop in HemSCs contributes to enhanced IH vasculogenesis in general.

### Notch pathway

The Notch pathway is a conserved ligand-receptor signaling mechanism that modulates cell fate and differentiation. The interaction of Notch receptors (Notch 1 to 4) with their ligands (Delta-like 1, -3, -4, Jagged-1 and -2) leads to the cleavage of the transmembrane Notch receptor, giving rise to the Notch intracellular domain (NICD) that migrates into the nucleus. In the nucleus, the NICD associates with a transcription factor, recombination signal binding protein for immunoglobulin kappa J (RBP-Jk), and activates transcription from the RBP-Jk DNA binding site. The NICD-RBP-Jk complex upregulates the expression of primary target genes of Notch signaling, such as hairy and enhancer of split (HES) and HES-related protein (HERP/HEY) family of transcription factors [54,55].

#### Notch expression in IH

Although the expression levels of the Notch components are likely dynamic during development, making transient expression difficult to detect, current data suggest that many known Notch components, mainly two ligands (Delta-like-4 and Jagged-1), three receptors (North-1, -3 and -4) and four effectors (HES-1, HEY1, HEY2 and HEYL) are involved in the pathogenesis of IH. Both Jagged-1 and Notch-4 are increased in proliferating IHs. All transcript levels of Notch-1, Notch-3, Notch-4, Jagged-1 and Delta-like-4 (Dll4) were higher in the IH than in the placenta (a commonly used tissue for comparisons). Conversely, Notch-2 is strongly decreased in both proliferating and involuting IHs [44,56].

#### Notch signaling triggers cell-cell interactions in IH

Notch signaling is initiated when the extracellular domain of the receptor engages ligands found on neighboring cells that are in close proximity to one another. Thus, Notch signaling depends on cell-contact-dependent interactions. In many cases, the cell that presents the ligand is a cell that does not have Notch signaling present, thus distinguishing two neighboring cells into one with ligand with little Notch signaling and one with receptor and high Notch signaling [57]. In a study by Wu et al. [44], the investigators demonstrated that HemSCs have distinct Notch expression patterns from HemECs. In HemSCs, where Notch3 is strongly expressed, HES1, HEY1, and HEYL were expressed at levels 10 to 100 times to that of HEY-2. In HemECs, however, Notch-1, Notch-4 and Jagged-1 have higher expression levels. HEY-2 proteins were often found to be expressed in HemECs. However, HEY-2 was not uniformly present in all ECs, suggesting that only a subset of IH ECs express the Notch target [56]. These data suggest the possibility that the Notch pathway might also contribute to establishing two distinct subpopulations at different steps of angiogenesis in IH, such as ECs versus smooth muscle cells (SMCs)/pericytes, arteries versus veins and large vessels versus capillaries [54,58,59]. We highlight the concept that ligand-receptor interactions in Notch signaling depend on contact between two cells, which may be two different cell types. Notch ligands involved in IH angiogenesis may be presented by HemECs, pericytes or HemSCs. Interestingly, research by Boscolol et al. [43] revealed that endothelial-derived Jagged-1 can induce HemSCs to acquire a pericyte-like phenotype, which is a crucial step in the vasculogenesis of IH. Disruption of the juxtacrine interaction between endothelial Jagged-1 and Notch receptors on HemSCs inhibited blood vessel formation in IH murine models. However, mice homozygous for a null mutation of several components of the Notch pathway, including Notch-1 and Jagged-1, resulted in embryonic lethality with vascular remodeling defects. Vasculogenesis proceeded normally in these mutants, whereas the next step, angiogenesis, was disrupted [60,61]. These data suggest that the upregulated Jagged-1 expression in the IH endothelium may provide a unique effect to control the vascular development of IH.

#### Is there a specific relationship between VEGF and Notch pathways in IH?

**Figure 4:**
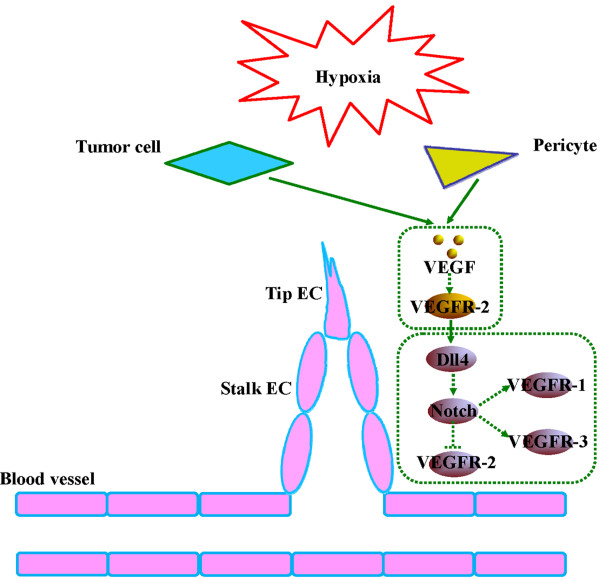
**Tip/stalk cell specification during spouting angiogenesis and vascular development.** Angiogenic sprouts emerge from the newly formed vessels in response to pro-angiogenic cues, such as hypoxia-induced VEGF. VEGF stimulus, acting via VEGFR-2, increases the expression of Dll4 on endothelial cells, which in turn activates Notch receptors on adjacent endothelial cells. Furthermore, VEGFRs are regulated by Notch signaling, providing an additional feedback loop between the two pathways: activated Notch receptors on ECs can positively regulate the expression of VEGFR-1 and VEGFR-3 in those cells. In contrast, Notch activation leads to the reduction of VEGFR-2 expression in cell culture and a concomitant decrease in the proangiogenic response to exogenous VEGF. Both of these effects would likely lead to a lower migratory or proliferative response in connector cells that exhibit Notch activation.

In vivo and in vitro studies have revealed several ways in which the VEGF and Notch pathways interact. Particularly, VEGF increases Dll4 expression [62,63]. Dll4 is strongly expressed by the ECs of sprouting angiogenic vessels, which commonly respond to VEGF signals. There is evidence that the blockade of VEGF in tumors results in a rapid decrease of Dll4 expression in tumor ECs [64]. Interestingly, although HemECs had higher VEGF-A levels and increased activation of VEGFR-2 compared with normal ECs [40], the Dll4 levels in HemECs were lower than those found in normal ECs [44]. These data argue against the idea that VEGF interacts with Notch signaling in IH. However, several lines of evidence indicate otherwise. For example, the disruption of Dll4 or endothelium-specific loss of Notch1 increases the superficial plexus vascular density and causes an excess of angiogenic sprouts. This loss of Notch signaling is associated with an increase in VEGFR-2 activity [57]. Other studies have suggested that reduced Notch activity resulted in reduced VEGFR-1 expression and increased VEGFR-2 expression in cultured ECs [65,66]. In addition, Hellstrom et al. [67] demonstrated that Dll4-Notch signaling within the endothelial cell population serves to suppress the tip-cell phenotype. The retinal vascular abnormalities in Dll4^+/-^ mice and after long-term treatment with γ-secretase inhibitors might also result from changes in the pattern of VEGF-A expression [67]. In contrast to Dll4, Jagged-1 is proangiogenic protein that functions by downregulating Dll4-Notch signaling. Jagged-1 also counteracts Dll4-Notch signaling interactions between stalk ECs, which helps to sustain elevated VEGF receptor expression in the newly formed and therefore immature vascular plexus at the angiogenic front [68]. By analogy to studies of VEGF signaling in HemECs, Notch components may be novel regulators for VEGF signaling in IH (Figure 4).

Until now, the involvement of Notch in IH development has remained poorly understood, and many issues still need to be addressed. How does the Notch pathway play a role in the interaction between HemECs and SMCs/pericytes? How are the different roles that the Notch pathway plays, such as arteriovenous patterning, tip cell differentiation and vessel wall formation, integrated during vascular development in IH? How do some of the key downstream Notch target genes affect IH vessels in the presence of high VEGF levels? And finally, does the expression and/or activity of VEGF components in IH depend on the nature of Notch signaling or vice versa? These questions should be addressed by future research efforts.

### β-adrenergic signaling

**Figure 5:**
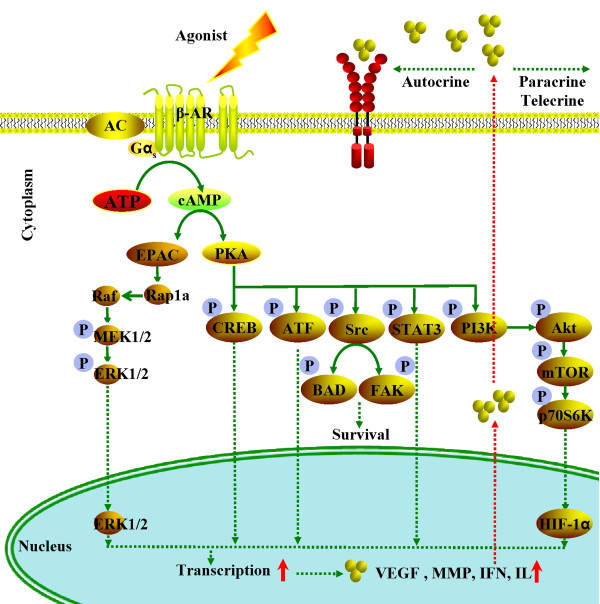
**β-adrenergic signaling modulates multiple cellular processes in tumor progression and metastasis.** The ligation of β-ARs by epinephrine or norepinephrine triggers a G-protein coupled signaling cascade that stimulates cAMP synthesis. cAMP activates the PKA protein, which can mediate multiple signal pathways via the phosphorylation of various downstream signal proteins. In another major pathway, the cAMP activation of EPAC leads to the Rap1A-mediated activation of Raf/MAPK signaling pathways and downstream effects on diverse cellular processes.

The β-adrenergic receptors (β-ARs), a family of G-protein-coupled receptors that are activated by β-adrenergic agonists (e.g., epinephrine or norepinephrine), can initiate a series of signaling cascades, thereby leading to multiple, cell-specific responses (Figure 5). The ligation of β-ARs by β-adrenergic agonists triggers a G-protein coupled signaling cascade that stimulates cyclic AMP (cAMP) synthesis. This secondary messenger, cAMP, regulates many cellular functions through its effectors, such as cAMP-dependent protein kinase (PKA) and EPAC (exchange proteins directly activated by cAMP) [69-71]. Preclinical studies have demonstrated that β-adrenergic signaling can regulate multiple fundamental biological processes underlying the progression and metastasis of tumors, including the promotion of inflammation [72-74], angiogenesis [75-78], migration [79], invasion [80,81] and resistance to programmed cell death [82-85]. Some evidence suggests that the stimulation of β-adrenergic signaling can also inhibit DNA damage repair and the cellular immune response [86,87] and promote surgery-induced metastasis [88,89]. These findings have led to the hypothesis that commonly prescribed β-blockers may favorably impact cancer progression and metastasis in patients [90].

In the six years since June 2008 when Leaute-Labreze et al. [11] first described their serendipitous observation of the anti-proliferative effect of propranolol on severe IHs, many articles regarding β-blocker therapy for IHs have been published [8,10,91]. However, despite the apparent widespread use of β-blockers, their mechanism of action in IHs has not yet been elucidated. Agonists and antagonists of β-ARs are known to act antithetically via the same intracellular pathways [92]. Given that the expression of all three β-ARs has been demonstrated in IH tumors [93-96], does β-adrenergic signaling play a role in the pathogenesis of IH? This hypothesis was immediately and, to some degree, indirectly testable by Mayer et al. [97], who found that intrauterine exposure to β_2_-sympathomimetic hexoprenaline can increase the occurrence of IH in preterm infants, suggesting a role for β-AR stimulation in the initiation of IH. Furthermore, we recently demonstrated that the activation of β-ARs resulted in increased HemEC proliferation and upregulation of the ERK signaling cascade. VEGFR-2-mediated ERK signaling was also upregulated upon β-AR activation to mediate the proliferation of HemECs [96]. These findings unveil a functional connection between the β-ARs and IH development. However, confirmatory studies in animal models of IH and mechanistic studies are needed to clearly define the role of β-adrenergic signaling in the growth and involution of IH [98].

### Tie-2/Angiopoietin signaling

Tie-2 and the angiopoietins (Ang), another receptor-ligand system involved in physiological and pathogenic angiogenesis, have also been reported to be associated with the development of IHs. Tie-2 tyrosine kinase receptor is expressed specifically on vascular ECs and on a certain subtype of macrophages implicated in angiogenesis. Ang-1 and Ang-2 have been identified as bona fide ligands of the Tie-2 receptor. Ang-1, which is mainly expressed by pericytes, is a critical player in vessel maturation and mediates the migration, adhesion and survival of ECs. Only tetrameric or higher multimeric forms of Ang-1 activate Tie-2, whereas oligomeric Ang-2 is a weak context-dependent agonist of Tie-2 and may even antagonize the receptor [99]. Ang-1-mediated Tie-2 activation stimulates a number of intracellular signaling pathways, such as the PI3K/Akt pathway, which promotes EC survival and nitric oxide (NO) synthesis by the activation of the mitogen-activated protein kinase (MAPK) pathway [100,101]. The deletion of Ang-1 between E10.5 and E12.5 results in an enlargement of vessel diameter, mainly in the capillaries [102,103]. Its phenotypes were comparable with those of IH, i.e., increased numbers of EC and overly covered by pericytes [25].

Using laser capture microdissection, Calicchio et al. [52] found Ang-2 was significantly increased in the IH endothelium compared with the placental vessels. In contrast, Ang-1 was decreased in proliferating IH relative to the placenta. Yu et al. [104] demonstrated that Tie-2 was specifically increased in HemECs and that this increase corresponds to enhanced cellular responses to the Tie-2 agonist Ang-1. Consistent with these findings, Boscolo et al. [42] revealed that hemangioma-derived pericytes exhibited low levels of Ang-1, resulting in a diminished ability to stabilize blood vessels in IH.

### HIF-α-mediated pathway

Hypoxia is one of the most powerful inducers of angiogenesis and vasculogenesis. During tumorigenesis, when tumor cells outgrow the limiting diffusion distance to nearby blood vessels and become hypoxic, the balance between pro-angiogenic and anti-angiogenic molecules is tipped towards pro-angiogenic molecules. This angiogenic switch provokes the expression of a variety of angiogenic factors by tumor cells and stromal cells, including VEGF-A, stromal cell-derived factor-1α (SDF-1α), fibroblast growth factor (FGF), platelet-derived growth factors (PDGFs), lysophosphatidic acid (LPA) and Ang [105].

Although the initiating mechanism during the pathogenesis of IH has yet to be discovered, there is evidence that tissue hypoxia may contribute to their explosive growth. The initial clinical description of the promontory mark of IH as an 'area of low blood flow’ suggests that tissue ischemia, a powerful stimulus for neovascularization, may be involved [106]. The hypothesis that ischemia/hypoxia plays a crucial role is also supported by the clinical observation of a blanched area of skin in the position of the future hemangioma. This region may be an area of local ischemia in the skin, caused by some unknown events, that creates a hypoxic environment and thus triggers growth factor expression.

In keeping with the observations described above, Ritter et al. [107] proposed a mechanism for myeloid cell-facilitated IH growth involving the hypoxia-induced expression of several growth factors (e.g., insulin-like growth factor-2) that drive endothelial proliferation. Kleinman et al. [108] demonstrated the presence of hypoxia-induced mediators of progenitor/stem cell trafficking in proliferating IH specimens and revealed that the combination of hypoxia and estradiol results in a synergistic effect on the upregulation of matrix metalloproteinase (MMP-9) in ECs in vitro, a key factor in endothelial progenitor cells (EPCs). The transcription factor hypoxia inducible factor (HIF-1α) was also stabilized in proliferating hemangioma specimens. Subsequent investigations revealed that HemECs show significantly a higher expression of HIF-1α than normal ECs. This upregulated HIF-α is a major contributor to the elevated VEGF levels produced in HemECs, and the decreased expression of HIF-α reduces the proliferation of these cells [39]. Moreover, the benefit observed during IH treatment by propranolol has been suggested to also be primarily due to the reduction of HIF-1α expression [109]. This suggestion has been confirmed by Chim et al. [110], who demonstrated that propranolol exerts its suppressive effects on HemECs through the HIF-1α-VEGF-A angiogenesis axis, the effects of which were mediated through the PI3/Akt and p38/MAPK pathways. Altogether, these findings indicate a direct and causative association between HIF-α signaling and the development of IH.

An additional possible effect of HIF-1α signaling in the pathogenesis of IH is mediation of EC autophagy. In their recent study, Chen et al. [111] revealed that a short exposure to hypoxia can induce HIF-α/BNIP3-dependent autophagy, which may promote EC survival growth. In contrast, if the hypoxia stress is prolonged, the autophagy activation may in turn become 5′-AMP-activated protein kinase (AMPK)/mammalian target of rapamin (mTOR) dependent and therefore cause programmed EC death. However, the evidence from this study was weakened by not being performed in IH-derived cells or in IH animal models.

### PI3K/Akt/mTOR signaling

PI3K generates 3-phosphorylated inositol lipids, causing the activation of downstream signaling, resulting in the activation of protein kinase B (PKB; also called c-Akt), which regulates, among others, mTOR, glycogen synthase kinase-3β and Forkhead box O transcription factor activity. Downstream targets of mTOR include p70 ribosomal protein 6S kinase (S6K). The overactivation of the PI3K/Akt/mTOR pathway, a signaling pathway that plays a key role in cellular growth and survival, has been implicated in various tumor pathogeneses, and as such, the inhibition of the PI3K/Akt/mTOR pathway is of therapeutic interest [112-114]. Medici and Olsen [39] found that HemECs had constitutively active PI3K/Akt/mTOR/p70S6K and tested their hypothesis that these cells could be sensitive to mTOR inhibitors (e.g., rapamycin). Finally, they demonstrated that the treatment of HemECs with rapamycin results in a significant decrease in HIF-1 and VEGF-A levels and in reduced proliferation. Strikingly, in vivo and in vitro studies further demonstrated that rapamycin can reduce the self-renewal capacity of the HemSCs, diminish the differentiation potential and inhibit the vasculogenic activity of these cells in vivo [115]. These preclinical data provide us with a pharmacological basis for the potential use of rapamycin in β-blocker-resistant IHs. Nonetheless, the mechanism that accounts for the effects of rapamycin in IH is far from clear, and a growing list of side effects make it doubtful that rapamycin would ultimately be beneficial in pediatric patients [112].

### PDGF-B/PDGFR-β signaling

The first evidence for a possible regulatory role of PDGF-B/PDGF receptor-β (PDGFR-β) signaling in IHs was provided by Walter et al. [116]. These researchers established a genetic linkage with chromosome 5q in three familial hemangiomas. The region, 5q31-33, contains three candidate genes involved in blood vessel growth. These genes were fibroblast growth factor receptor-4 (FGFR4), PDGFR-β and VEGFR-3 [116]. Subsequently, a study examining global gene expression changes between the IH growth phases by the genome-wide transcriptional profiling of blood vessels showed a reduction in PDGFR-β expression during the involutive phase [52]. These findings provide the possibility that PDGF-B/PDGFR-β signaling may play a role in IH pathogenesis.

The endothelium is a critical source of PDGF-B for PDGF-β-positive mural cell recruitment, as demonstrated by the endothelium-specific ablation of PDGF-B, which leads to pericyte deficiency [117]. The blockade of pericyte recruitment by abolishing PDGF-B/PDGFR-β signaling causes a lack of basement membrane matrix deposition and concomitantly increased vessel widths [118]. In addition, the ectopic expression of PDGF-B by tumor cells results in the increased recruitment of mural cells to blood vessels on the establishment of subcutaneous tumors [119,120]. Unfortunately, despite the tight physical and functional association between ECs and pericytes, there is a paucity of information about the signals exchanged between the two cell types in IHs. Reassuringly, data from a separate study demonstrated that PDGF/PDGF-R-β signaling may act as an intrinsic negative regulator of IH involution. In this study, Roach and colleagues [121] found that PDGF is elevated during the proliferating phase and may inhibit adipocyte differentiation. The exposure of HemSCs to exogenous PDGF results in an activation of autocrine PDGF/PDGF-R-β signaling, thereby inhibiting IH involution. These findings highlight the involvement of PDGF/PDGF-R-β signaling in the development of IHs. Moreover, hemangioma-derived pericytes also express PDGFR-β, although its effect has not been elucidated in IH pathogenesis [42]. Thus, the possibility of targeting HemSC and Hem-pericyte function, for example, via their PDGF receptors, to gain enhanced efficacy of antiangiogenic treatment regiments is supported by the reports of beneficial effects of combining PDGFR inhibitors with antiangiogenic drugs or regimens [118,122,123].

### Conclusion and future challenges

In conclusion, the findings summarized above demonstrate that signaling pathways involved in the development of IH are increasingly being clarified, underscoring their significant relevance to understanding IH pathogenesis. However, similar to malignant tumors, there is extensive crosstalk between individual signaling pathways in IH. This crosstalk is generally due to two factors. First, multiple pathways often control a common process. Second, many signaling outcomes impact other processes through feedback loops and compensatory responses. Therefore, elucidating the molecular pathogenesis of IH presents an intriguing challenge. To solve this puzzle, an organized reconstruction of the sequential molecular perturbations during IH neovascularization is required. Such an analysis needs to combine data from different levels, including genetic aberrations, expression alterations and protein modification in a comprehensive set of tissue samples. These issues should highlight the important role that the increased knowledge of the molecular pathways involved in the pathogenesis of IH will have in guiding the development of effective, rationally designed therapeutic strategies. Future research efforts will not only provide us with a pharmacological basis of the therapeutic use of β-blocker in IHs but also a basis for the further investigation of other potential anti-hemangioma agents.

## Competing interests

The authors declare that they have no competing interests.

## Authors’ contributions

YJ and SYC drafted the manuscript. All the authors have read and approved the final manuscript.
